# Multiple Calcifying Hyperplastic Dental Follicle (MCHDF): A Case Report

**DOI:** 10.5681/joddd.2013.028

**Published:** 2013-08-30

**Authors:** Shokoofeh Jamshidi, Massoumeh Zargaran, Nooshin Mohtasham

**Affiliations:** ^1^Department of Oral and Maxillofacial Pathology, Faculty of Dentistry, Hamadan University of Medical Sciences, Hamadan, Iran; ^2^Dental Research Center ,Department of Oral and Maxillofacial Pathology, Faculty of Dentistry, Hamadan University of Medical Sciences, Hamadan, Iran; ^3^Department of Oral and Maxillofacial Pathology, Faculty of Dentistry, Mashhad University of Medical Sciences, Mashhad, Iran

**Keywords:** Dental follicle, hyperplastic, teeth

## Abstract

Evaluation of enlarged follicles with unerupted teeth is always important because some changes may occur. One of the ex-tremely rare conditions seen in dental follicles is multiple calcifying hyperplastic dental follicle. We report a case of multi-ple calcifying hyperplastic dental follicle. Radiographs showed that mandibular third molars had a pericoronal radiolucent zone delineated by a well-defined and sclerotic border. Microscopic examination revealed a combination of fibrous connec-tive and small calcifications. Multiple calcifying hyperplastic dental follicle is a rare condition and its correct diagnosis is necessary to apply appropriate treatment.

## Introduction

Evaluation of enlarged follicles with unerupted teeth is always important because some changes (such as cystic or tumoral) may occur.^1^ One of the extremely rare conditions seen in dental follicles is multiple calcifying hyperplastic dental follicle (MCHDF).^2^ Many authors have suggested that this condition could be considered a pathologic entity because it is sufficiently distinctive.^3,4^ The term MCHDF, first described by Sandler et al, represents a dental follicle of an unerupted tooth with numerous calcifications and rests of odontogenic epithelium.^2^

## Case report

**Figure 1:**
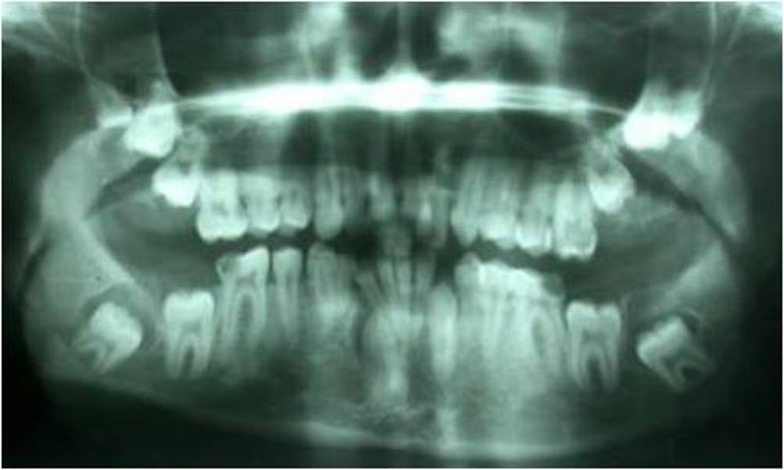
Radiograph illustrates 12 embedded teeth.

A 19-year-old white man, with a chief complaint of unerupted teeth, was referred to Faculty of Dentistry, Hamadan University of Medical Sciences, in December 2008. The panoramic radiograph showed that the left and right second and third molars, maxillary distomolars, and left and right mandibular canines were unerupted. This radiograph showed that mandibular third molars had a pericoronal radiolucent zone delineated by a well-defined and sclerotic border (Figure 1).

These circumcoronal radiolucencies were approximately 11 mm in diameter on the right and 12 mm in diameter on the left side. The patient had no medical history. After surgery, mandibular third molars with their follicles were sent for pathologic examination. Grossly, the material of right side consisted of one soft tissue specimen, 10×6×3 mm in size, and on the left side it consisted of two soft tissue specimens, one 14×6×2 mm and the other one 8×6×5 mm in size, with a white-yellow color.

Microscopic examination showed that fibrous connective tissue was composed of fibroblasts, collagen fibers, odontogenic epithelium rests and small calcifications, similar to type A calcifications in the hyperplastic follicle of regional odontodysplasia. In many areas these calcifications fuse to one another and make larger bodies. In addition, type B calcification was seen around fibrillar tufts, which is identical to regional odontodysplasia.

## Discussion

**Figure 2:**
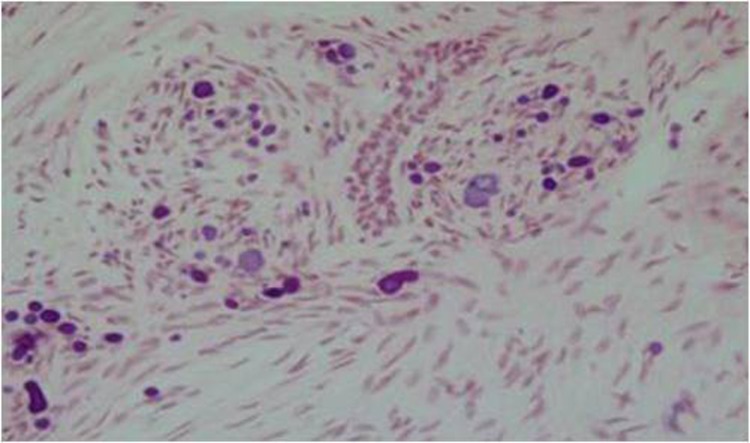
Dental follicles showing cellular connective tissue.

To date, 10 cases of MCHDF have been published worldwide.^5^ All the patients have been males, with the affliction of the mandible in most cases.^6^ Our case was the 11th example of MCHDF and similar to previous cases the patient was male and the mandible was affected. Histologically, MCHDF resembled WHO type of central odontogenic fibroma. Both lesions have connective tissues, rests of odontogenic epithelium and calcifications. The connective tissue of odontogenic fibroma (WHO type) is very cellular and often interwoven with less cellular areas, while it might be quite vascular.^3^ However, our case lesions consisted of relatively dense, moderately cellular, fibrous connective tissue (Figure 2).

Moreover, WHO type of central odontogenic fibroma exhibits calcifications composed of cementum-like material or dentinoid.^7^ In MCHDF the calcified materials are classified as:

Type I) acellular and relatively homogenous calcified matrices

Type II) cellular calcification, mostly resembling woven bone^5^

In addition, some studies have described histopathologic pattern of MCHDF calcifications as type A (calcification arranged in whorled structure) and type B (calcification with peripheral tufts within fibrous connective tissue stroma).^3^

**Figure 3:**
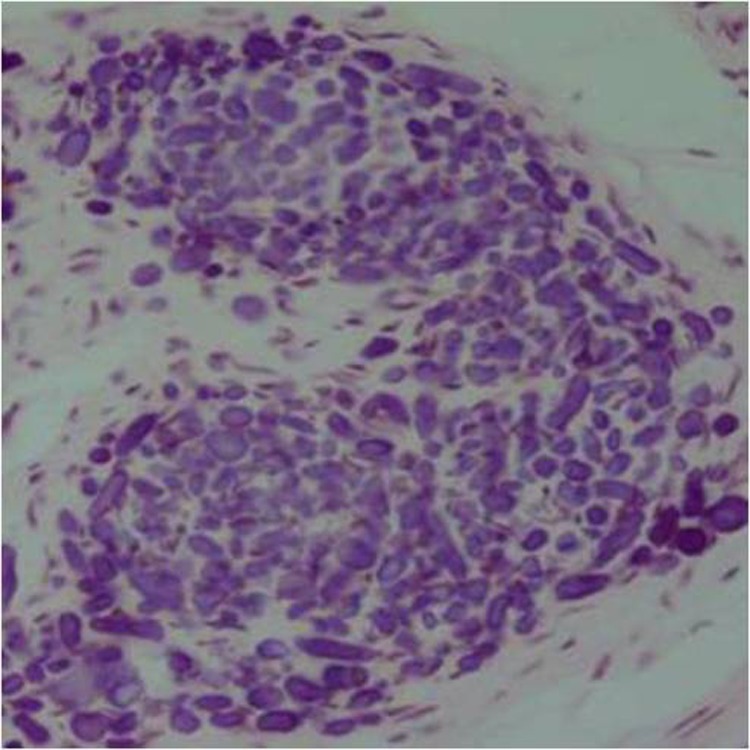
Type A calcification arranged in whorled structures(hematoxylin-eosin stain, ×400).

**Figure 4:**
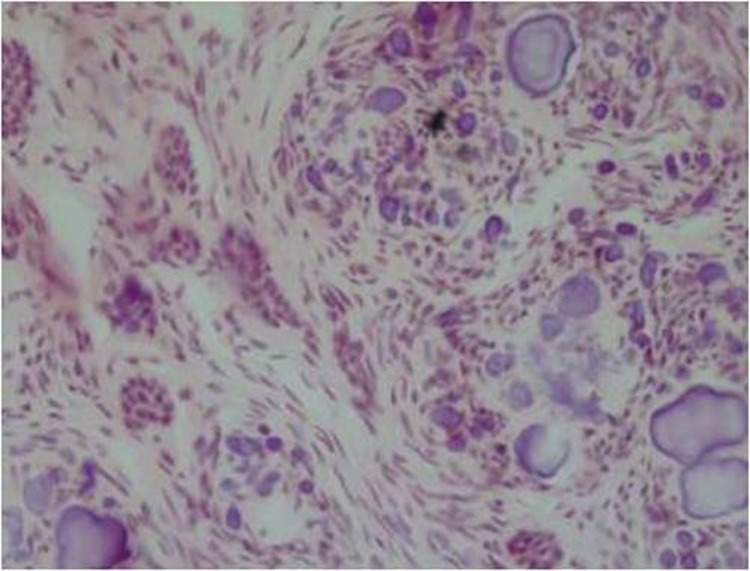
Type B calcification within cellular connective tissue stroma (hematoxylin-eosin stain, ×400)

In our case, histopathologic pattern of calcification was type A and B (Figures 3 and 4). Similarly, Gardner and Radden, along with Gomez et al, reported two types of calcifications, whereas only type A calcification was observed by Sandler et al and Lukinmaa et al.^2-4,6^

Despite a report by Gomez et al, our case did not have any islands of clear odontogenic epithelium, although it exhibited rests of odontogenic epithelium in connective tissue.^4^

MCHDF is histologically identical to the hyperplastic dental follicles of regional odontodysplasia, but malformations of teeth that are seen in regional odontodysplasia were not found in MCHDF.^3,4,8^ Domingues et al described a new entity of fibro-odontogenic dysplasia that exhibited histologic similarity to MCHDF, but the former shows osseous development and a familial trait.^4,9^ In MCHDF multiple unerupted teeth occur, similar to cleidocranial dysplasia and Gardner's syndrome, although there is no relationship between MCHDF and these disorders.^3^ To date, the etiology of MCHDF is not known, but as suggested by previous authors, these condition could be considered as one pathologic entity with the term multiple calcifying hyperplastic dental follicle.^2-4,6^
